# Lipoprotein subfractions in cardiovascular disease risk prediction and therapeutic targeting: Current landscape and future directions

**DOI:** 10.1515/jtim-2026-0025

**Published:** 2026-04-18

**Authors:** Long Zhang, Shuo Zhang, Yangyang Sun, Leyi Wang, Fangfang Fan, Yan Zhang, Jianping Li

**Affiliations:** Department of Cardiology, Peking University First Hospital, Beijing, China; Institute of Cardiovascular Disease, Peking University First Hospital, Beijing, China; State Key Laboratory of Vascular Homeostasis and Remodeling, Peking University, Beijing, China

Dyslipidemia is a well-established driver of atherosclerotic cardiovascular disease (ASCVD). However, substantial residual risk persists despite aggressive lowering of low-density lipoprotein cholesterol (LDL-C). Advances in analytical techniques have strengthened the scientific consensus that specific lipoprotein subfractions contribute to this residual risk.^[1]^

## Lipoprotein subfractions associated with ASCVD

### LDL Subfractions

Current research has increasingly focused on LDL subfractions such as particle size, particle concentration, and oxidative modification. As for LDL size, pathological researches indicate that small dense LDL (sdLDL) particles may exhibit heightened atherogenicity, attributed to their enhanced ability to infiltrate the arterial intima, increased susceptibility to oxidation, and prolonged circulation time. Nevertheless, in clinical researches, the association between sdLDL and ASCVD risk is substantially attenuated or nullified after adjustment for conventional risk factors,^[2]^ And the Mendelian randomization (MR) analysis revealed that, after adjusting for triglyceride (TG), high density lipoprotein cholesterol (HDL-C), and LDL-C, sdLDL had no significant impact on myocardial infarction (MI). Thus, the evidence regarding the connection between LDL size and cardiovascular events remains inconclusive.^[3]^ As for LDL particle (LDL-P) concentration, in Framingham Offspring Study (a large community-based sample consist of middle-aged white participants), LDL-P showed a stronger association with ASCVD than non-HDL-C (HR: 1.28 *vs*. 1.21). In contrast, the Multi-Ethnic Study of Atherosclerosis found no incremental predictive value for LDL-P. MR analysis indicated that, in the univariable analysis, both large LDL-P and small LDL-P showed potential causal associations with the incidence of MI. However, these associations did not remain statistically significant after adjusting traditional lipid risk factors.^[3]^ Therefore, current evidence remains insufficient to establish a causal relationship between LDL-P and cardiovascular events. As for LDL condition, oxidized LDL (oxLDL) contributes to atherosclerosis *via* endothelial dysfunction, monocyte recruitment, macrophage polarization, and plaque destabilization. Several Meta analyses have confirmed that oxLDL is elevated in cardiovascular disease (CVD) patients, supporting its role in cardiovascular risk. The oxidized LDL receptor 1 has thus emerged as a promising immunotherapeutic and drug delivery target, with recent evidence indicating its potential to reduce non-calcified plaque burden in diabetics participants.^[4]^ Therefore, oxLDL may serve as a novel biomarker within the LDL subfractions, offering enhanced predictive value for residual cardiovascular events.

### HDL subfractions

The conventionally measured HDL-C level has proven to be an incomplete predictor of cardiovascular risk. One explanation is that impaired HDL function—such as in reverse cholesterol transport (RCT), anti-inflammatory, antioxidant, and antithrombotic activities—may offset the potential benefits of HDL-C. Consequently, other biomarkers such as HDL-P number and size may be better indicators for HDL function. As for particle numbers, several prospective cohort studies have indicated that HDL-P is inversely associated with the risk of ASCVD, MI, and stroke, independent of HDL-C and metabolic confounders.^[5]^ The MR analysis demonstrated that a higher number of both medium and small HDL-P was associated with a reduced risk of MI, independent of traditional risk factors.^[3]^ This finding reinforces the protective role of HDL-P. However, clinical application of HDL-P remains limited, partly due to a lack of interventional evidence showing that modulating HDL-P could improves outcomes. As for particle size, HDL is commonly divided into larger, lipid-rich HDL2 and smaller, dense HDL3. These subclasses exhibit distinct functional properties: HDL2 shows strong efficacy in RCT and anti-inflammatory activity, whereas HDL3 promotes cholesterol efflux and carries negatively charged phospholipids that confer potent antioxidant, antithrombotic, and anti-apoptotic effects. Nevertheless, conflicting epidemiological data persist. Some population studies^[6]^ suggest HDL2 is more cardioprotective, while others report a stronger inverse association between HDL3 and cardiovascular risk. These discrepancies may be compounded by methodological variations and population-specific.^[7]^ MR analysis indicates that a larger HDL-P size is associated with an increased risk of MI, in contrast to the protective effect of small HDL particles.^[3]^ This underscores the current consensus on the protective role of small HDL particles. Therefore, it is essential to re-evaluate the association between HDL particle size and cardiovascular events within a standardized lipid testing framework. Finally, Apolipoprotein A1 (ApoA1), a key component of HDL, facilitates cholesterol efflux from macrophages, which is negatively correlated with cardiovascular risk. The large-scale randomized controlled trial AEGIS-II evaluated the long-term (1-year) prognosis of patients receiving weekly intravenous infusions of 6 g CSL112 (human ApoA1). The primary results showed no significant improvement in major adverse cardiovascular events (MACE) compared to placebo.^[8]^ However, further analysis revealed that among patients with baseline LDL-C ≥ 100 mg/dL or persistent high-risk factors, CSL112 infusion was associated with a reduced risk of recurrent cardiovascular events.^[9,10]^ This suggests that ApoA1-based intervention therapy still holds promise.

### Triglyceride-rich lipoprotein (TRLs)

Currently, numerous studies are focused on elucidating the relationship between TG and TRLs in the context of cardiovascular disease. TRLs comprise a heterogeneous group of lipoprotein particles—such as remnant lipoprotein-cholesterol (RLP-C) and sdLDL—that originate from intestinal chylomicrons or hepatic very-low-density lipoprotein (VLDL).^[11]^ Accumulating evidence supports a robust association between TRLs and ASCVD. For example, a biracial cohort analysis from the Jackson Heart Study and the Framingham Offspring Cohort reported a positive correlation between RLP-C and CVD incidence in unadjusted models. Similarly, the Women’s Health Study recently identified a close relationship between TRLs and future incidents of MI and peripheral artery disease. Similarly, a large-scale Mendelian randomization study involving 958,434 participants provided robust evidence that elevated RLP-C levels are associated with significantly increased risks of CVD, MI, and stroke, independent of LDL-C. Moreover, genetic studies on related drug targets support the potential cardiovascular benefit of lowering RLP-C.^[12]^ In parallel, clinical intervention studies are exploring whether modulating TRLs can improve outcomes. The REDUCE-IT randomized controlled trial demonstrated that treatment with icosapent ethyl (IPE) effectively lowered TG and RLP-C, which subsequently reduced the risk of cardiovascular events.^[13]^ Although fibrates also lower TG levels, they have not demonstrated consistent benefits on ASCVD outcomes. Emerging therapeutic strategies—such as gene-silencing agents targeting APOC3 and ANGPTL3—offer novel approaches for managing hypertriglyceridemia, showing potent TG-lowering efficacy and a favorable safety profile.^[14]^ Nevertheless, further investigation is still required to determine whether TG-lowering interventions consistently influence cardiovascular outcomes (Table 1).

### Lipoprotein (a) (Lp [a] )

Lp (a) is now recognized as an important independent genetic risk factor for ASCVD. Lp (a) promotes atherosclerosis through several distinct mechanisms. First, similar to LDL, it facilitates cholesterol deposition within the arterial wall. Second, the unique structure of apolipoprotein (a) (apo [a] ) shares high homology with plasminogen, enabling Lp (a) to competitively inhibit fibrinolysis and promote thrombogenesis. Third, Lp (a) acts as a major carrier of oxidized phospholipids, which accumulate in the vascular wall and amplify inflammatory responses. Substantial epidemiological and genetic studies provide strong evidence supporting these mechanisms. Consequently, numerous contemporary guidelines now specify both the appropriate timing for Lp (a) testing and corresponding clinical target values.^[15]^ However, two major challenges currently limit the routine clinical use of Lp (a). The first is assay standardization; size polymorphism of apo (a) impairs comparability across different measurement methods, hindering the establishment of universal diagnostic cutoffs and consistent guideline implementation. The second is the lack of effective therapies. Conventional lipid-lowering agents such as statins have minimal effect on Lp (a), while PCSK9 inhibitors only modestly lower Lp (a) levels without specificity. Novel Apo (a) inhibitors have demonstrated potent and selective Lp (a) reduction in clinical trials. However, whether this translates into reduced cardiovascular events awaits confirmation from ongoing outcome trials^[16]^ (Table 1).

**Table 1:** The effects of lipid-lowering therapy on lipoprotein subfractions

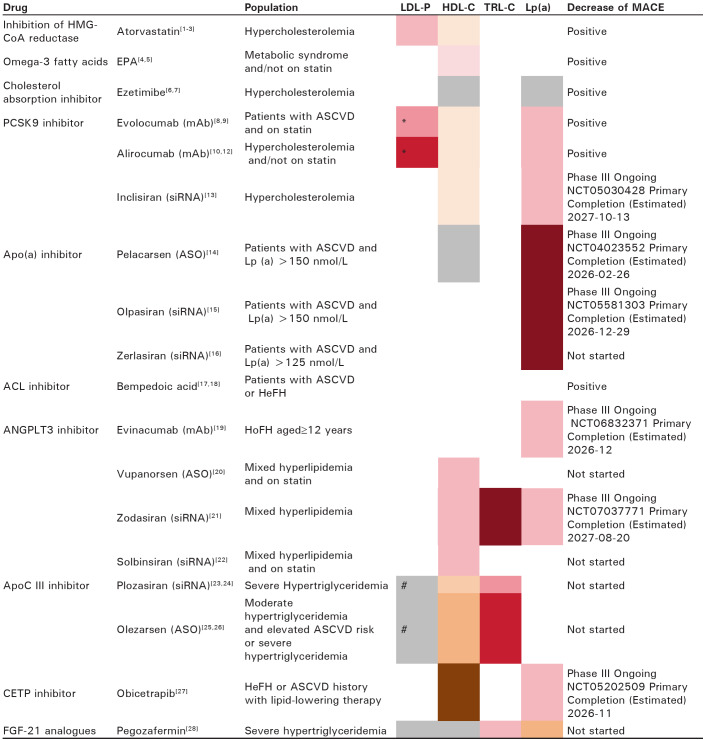

^*^ means decrease of both small and large LDL particles; # means increase of large LDL particles and decrease of small LDL particles. HeFH: heterozygous familial hypercholesterolemia; HoFH: homozygous familial hypercholesterolemia; ACL: adenosine triphosphate-citrate lyase; PCSK9: proprotein convertase subtilisin/kexin type 9; ANGPTL3: angiopoietin like 3; HMG-CoA: 3-hydroxy-3-methylglutaryl coenzyme A; EPA: eicosapentaenoic acid; FGF-21: fibroblast growth factor 21; CETP: cholesteryl ester transfer protein; LDL-P: low-density lipoprotein particle; Lp (a): lipoprotein (a); HDL-C: high-density lipoprotein cholesterol; TG: triglyceride; TRL-C: triglyceride-rich lipoproteins; mAb: monoclonal antibody; siRNA: small interfering RNA; ASO: antisense oligonucleotide. Mixed hyperlipidemia: fasting level of 150 to 499 mg/dL and either an LDL cholesterol level of ≥ 70 mg/dL or a non-HDL cholesterol level of ≥ 100 mg/dL; Severe HTG: TG ≥ 500 mg/dL; moderate HTG: TG level within 150 to 499 mg/dL. Detailed data and references could be found in Supplementary Table S1.

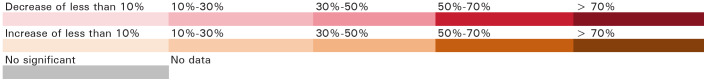

## Intervention strategies targeting lipoprotein subfractions

Currently, a growing number of therapeutic agents have been developed to precisely target specific subcomponents of pathogenic lipoproteins. Examples include Apo (a) inhibitors such as Pelacarsen, Olpasiran, and Zerlasiran for Lp (a) reduction; APOCIII inhibitors like Plozasiran and Olezarsen for TRLs. Phase II clinical trials for these agents have consistently demonstrated robust therapeutic efficacy in the targeted patient populations. Moreover, the latest lipid-lowering drug, FGF21 analog (pegolafrozin),^[17]^ could significantly reduce TRLs, but is associated with a mild increase in Lp (a) (Table 1 and Supplementary Table S1). Nevertheless, it remains necessary to await the outcomes of Phase III clinical trials to ascertain their impact on MACE. More importantly, with the discovery of an increasing number of novel lipid subcomponent biomarkers, future research will need to explore a broader range of lipid-lowering agents. This exploration is essential to achieve the dual goals of precise lipid modulation and reduction of ASCVD risk.

## Future directions

Although lipoprotein subfractions are strongly implicated in residual ASCVD risk, their clinical translation faces two major challenges. First, the absence of standardized measurement methodologies compromises cross-study comparability and underscores the need for more refined and harmonized assays. Fortunately, advances in analytical platforms—such as nuclear magnetic resonance (NMR) spectroscopy, vertical auto profile (VAP), and mass spectrometry—are progressively establishing robust subfraction profiling methods. Among these, VAP technology offers high precision, reproducibility, and cost-effectiveness, meriting broader research attention. Second, building on standardized lipid testing, more precise investigations are needed to elucidate how lipid-lowering drugs modulate specific lipoprotein subfractions. Future studies should confirm whether cardiometabolic drugs can improve cardiovascular outcomes by modulating atherogenic lipoprotein subfractions.

## Supplementary Material

Supplementary Material Details

## References

[1] Nordestgaard BG, Langlois MR, Langsted A, Chapman MJ, Aakre KM, Baum H (2020). Quantifying atherogenic lipoproteins for lipid-lowering strategies: Consensus-based recommendations from EAS and EFLM. Atherosclerosis.

[2] Hirano T (2025). Clinical significance of small dense low-density lipoprotein cholesterol measurement in type 2 diabetes. J Diabetes Investig.

[3] Zhao Q, Wang J, Miao Z, Zhang NR, Hennessy S, Small DS (2021). A Mendelian randomization study of the role of lipoprotein subfractions in coronary artery disease. Elife.

[4] Hernando-Redondo J, Niño OC, Fitó M (2025). Atherogenic low-density lipoprotein and cardiovascular risk. Curr Opin Lipidol.

[5] Kontush A (2015). HDL particle number and size as predictors of cardiovascular disease. Front Pharmacol.

[6] Piko P, Kosa Z, Sandor J, Seres I, Paragh G, Adany R (2022). The profile of HDL-C subfractions and their association with cardiovascular risk in the Hungarian general and Roma populations. Sci Rep.

[7] Zhang Y, Yu M, Chen Y, Huang J (2026). High-density lipoprotein in cardiovascular diseases: From high quantity to high quality. Clin Chim Acta.

[8] Gibson CMD, Duffy S, Korjian MC, Bahit G, Chi JH, Alexander AM (2024). Apolipoprotein A1 Infusions and Cardiovascular Outcomes after Acute Myocardial Infarction. N Engl J Med.

[9] Gibson CM, Chi G, Duffy D, Bahit MC, White H, Korjian S (2024). ApoA-I Infusions and Burden of Ischemic Events After Acute Myocardial Infarction: Insights From the AEGIS-II Trial. J Am Coll Cardiol.

[10] Gibson CM, Duffy D, Bahit MC, Chi G, White H, Korjian S (2024). Apolipoprotein A-I infusions and cardiovascular outcomes in acute myocardial infarction according to baseline LDL-cholesterol levels: the AEGIS-II trial. Eur Heart J.

[11] Duran EK, Aday AW, Cook NR, Buring JE, Ridker PM, Pradhan AD (2020). Triglyceride-Rich Lipoprotein Cholesterol, Small Dense LDL Cholesterol, and Incident Cardiovascular Disease. J Am Coll Cardiol.

[12] Navarese EP, Vine D, Proctor S, Grzelakowska K, Berti S, Kubica J (2023). Independent Causal Effect of Remnant Cholesterol on Atherosclerotic Cardiovascular Outcomes: A Mendelian Randomization Study. Arterioscler Thromb Vasc Biol.

[13] Bäck M (2023). Icosapent ethyl in cardiovascular prevention: Resolution of inflammation through the eicosapentaenoic acid - resolvin E1 - ChemR23 axis. Pharmacol Ther.

[14] Gomez-Delgado F, Raya-Cruz M, Katsiki N, Delgado-Lista J, Perez-Martinez P (2024). Residual cardiovascular risk: When should we treat it?. Eur J Intern Med.

[15] Duarte Lau F, Giugliano RP (2022). Lipoprotein(a) and its Significance in Cardiovascular Disease: A Review. JAMA Cardiol.

[16] Greco A, Finocchiaro S, Spagnolo M, Faro DC, Mauro MS, Raffo C (2025). Lipoprotein(a) as a Pharmacological Target: Premises, Promises, and Prospects. Circulation.

[17] Bhatt DL, Bays HE, Miller M, Cain JE, Wasilewska K, Andrawis NS (2023). The FGF21 analog pegozafermin in severe hypertriglyceridemia: a randomized phase 2 trial. Nat Med.

