# Supplementary material for: Lipoprotein subfractions in cardiovascular disease risk prediction and therapeutic targeting: Current landscape and future directions
Source: J Transl Int Med. 2026 Apr 18;14(3):350–4. doi: 10.1515/jtim-2026-0025 (PMC13320524; doi:10.1515/jtim-2026-0025)
Supplement: Supplementary file 1 — Supplementary Material Details [file jtim-2026-0025_sm.pdf]

Supplementary materials

Supplement Table S1: Current lipid-lowering treatments and their effects on lipoprotein subfractions.

Drug	Population	Time	Dose	Statistic Data	LDL-C related		HDL-C related		TRL-C related			Non-HDL-C	Lp (a)	Apoproteins	
					LDL-C	LDL-P and size	HDL-C	HDL-P	TG	VLDL-C	RC/TRL-C			ApoB	ApoA1
Atorvastatin[1-3]	HC	12 w	20mg qd	%mean change from base line	↓42.4%	↓30%	↑7.0%		↓4.1%			↓40.2%		↓36.2%	↑2%
EPA[4,5]	MS and on statin	12 w	4g qd	%mean change from base line	↓5.2%		↓4.0%		↓21.7%			↓13.5%		↓8.8%	
Ezetimibe[6,7]	HC	12 w	10mg qd	%mean change from base line	↓18.4%		↑4.2%; (HDL2-C ↑7.9%, HDL3-C ↑3.4%)		↓5.1%			↓17.7%	↑12.4%	↓15.4%	↑2.4%
Evolocumab (anti-PCSK9 mAbs)[8,9]	CAD and on statin	76 w	420mg qm	%mean change from base line	↓60.7%	↓44.1%; ↓large LDL particles, ↓small LDL particles	↑7%	↑9.4%	↓9.3%			↓52.1%	↓26%	↓49.6%	↑6%

Alirocumab (anti-PCSK9 mAb)[10-12]	HC and/not on statin	24 w	150 mg q4w,75 mg q2w,150 mg Q2W	placebo-adjust %mean change from base line	↓56.4-58%	↓63.3%; ↓large LDL particles, ↓small LDL particles	↑9.8-10.1%	↑11.2 %	↓10.4 -11.8%			↓49-50.1%	↓19.6-25.9%	↓46.4-47.2%	↑4.8-6.6%
Inclisiran (siRNA PCSK9 inhibitor)[13]	High risk for CVD and high LDL-C	180 d	200/300/500mg *1 or 100/200/300mg *2	%mean change from base line	↓27.9-52.6%		↑4.4-10.3%		↓6.3-14.2%	↓11.6 -23.8%		↓25.1 -46%	↓14.3-25.6%	↓22.9-40.9%	↑2.9-8.6%
Pelacarsen [Apo(a)ASO][14]	CVD and Lp(a) > 150 nmol/l	6 m	20/40/60mg q4w or 20mg q2w or 20mg qw	%mean change from base line	↓6-21%		↑0-6%		↓0-9%				↓38-80%	↓2.7-15%	
Olpasiran [Apo(a)siRNA][15]	ASCVD and Lp(a) > 150 nmol/l	36 w	10mg q12w or 75mg q12w or 225mg q12w or 225mg q24 w	placebo-adjust LSM % change	↓22.6%-23.7%								↓70%-101.1%	↓16.7-18.9%	
Zerlasiran [Apo(a) siRNA][16]	ASCVD and Lp(a) > 125	36 w	450mg q24w *2 or	placebo-adjust	↓25.1-31.9%								↓81.3-85.6%	↓9.9-15%	

	nmol/l		300mg q16w *3 or 300mg q24w *2	LSM % change						
Bempedoic acid[17,18]	ASCVD, HeFH, or LDL -C > 70 mg /dl and with maximum statin	12 w	180mg qd	placebo-adjust LSM % change	↓18.1%			↓13.3 %		↓11.9%
Evinacumab (anti-ANGPTL3 mAb)[19]	HoFH aged ≥12	24 w	15mg/kg q4w	%mean change from base line	↓41.3-47.8%		↓50.8 -51.8%	↓44.2 -49.6%	↓12.4-18.2%	↓34.6-41.2%%
Vupanorsen (ANGPLT3 ASO)[20]	Non-HDL-C≥100 mg/dL and TG 150 - 500 mg/dL on statin therapy	24 w	80/120/160 mg q4w or 60/80/120/160mg q2w	placebo-adjust LSM % change	↓7.9-16%	↓12.1-35.1%	↓41.3 -56.8%	↓22-27.7%		↓6-15.%

Zodasiran (ANGPLT3 siRNA)[21]	Mixed hyperlipidemia	24 w	50/100/200 mg q12w	placebo-adjust LSM % change	↓13.6-19.9%		↓12-24.5%		↓51.2-63.1%		↓72.6-82%	↓28.7-36.4%	↓7.3-20%	↓15.2-21.9%	
Solbinsiran (ANGPLT3 siRNA)[22]	Mixed hyperlipidemia and on statin	180 d	100mg/400mg/800mg q90d	placebo-adjusted % mean change	↓1.3-16.8%		↓6.1-22.7%		↓36.3-52.5%	↓41.6-50.1%		↓10.8-25.5%		↓2.8-14.3%	
Plozasiran (ApoCIII siRNA)[23,24]	SHTG	48 w	10/25/50mg q3m	placebo-adjust LSM % change	↑12.2-23.2%	↓4.2%; ↑ large LDL particles, ↓small LDL particles	↑17.5-31.8%	↑8-12%	↓24.9-51.5%		↓26.2-46.8%	↓10-23.5%		↓0-5.9%	
Olezarsen (ApoCIII ASO)[25,26]	MHTG or SHTG	6 m	50mg/80mg qm	placebo-adjust LSM % change	↓1.1-1.5%	↓3.8-6%; ↑ large LDL particles, ↓small LDL particles	↑45.5-47.6%	↑15%	↓58.4-60.6%	↓56.6-57.4%	↓66.1-68.4%	↓21.7-21.9%		↓14.7-14.9%	
Obicetrapib (CETP inhibitor)[27]	HeFH or ASCVD history and on LLT	84 d	10mg qd	placebo-adjust LSM % change	↓32.6%		↑136.3%		↓8%			↓29.4%	↓33.5%	↓18.9%	↑43.2%

Pegozafermin (with FGF-21 analogues)[28]	SHTG	8 w	9/18/27mg qd or 36mg q2w	% mean change from base line (integrated)	↑10%	↑ 4.5%	↑25%		↓57%	↓47.6% (VLDL-TG↓58.5%)	↓29.2%	↓18%	↑39.49%	↓11%;(ApoB48↓58.2%)

HeFH: heterozygous familial hypercholesterolemia; HoFH: homozygous familial hypercholesterolemia; ACL: adenosine triphosphate-citrate lyase; PCSK9: proprotein convertase subtilisin/kexin type 9; ANGPTL3: angiopoietin like 3; HMG-CoA: 3-hydroxy-3-methylglutaryl coenzyme A; EPA: eicosapentaenoic acid; FGF-21: fibroblast growth factor 21; CETP: cholesteryl ester transfer protein; LDL-P: low-density lipoprotein particle; Lp(a): lipoprotein(a); HDL-C: high-density lipoprotein cholesterol; TG: triglyceride; TRL-C: triglyceride-rich lipoproteins; mAb: monoclonal antibody; siRNA: small interfering RNA; ASO: antisense oligonucleotide; HC: hypercholesterolemia; MS: metabolic syndrome; CAD: coronary atherosclerosis disease; CVD: cardiovascular disease; ASCVD: atherosclerotic cardiovascular disease; MHTG: moderate hypertriglyceridemia; SHTG: severe hypertriglyceridemia; LLT: lipid-lowering therapy. Mixed hyperlipidemia: fasting level of 150 to 499 mg/dL and either an LDL cholesterol level of ≥70 mg/dL or a non-HDL cholesterol level of ≥100 mg/dL; Severe HTG: TG ≥500 mg/dL; moderate HTG: TG level within 150 to 499 mg/dL.

References:

1. Colhoun HM, Betteridge DJ, Durrington PN, Hitman GA, Neil HA, Livingstone SJ, Thomason MJ, Mackness MI, Charlton-Menys V and Fuller JH. Primary prevention of cardiovascular disease with atorvastatin in type 2 diabetes in the Collaborative Atorvastatin Diabetes Study (CARDS): multicentre randomised placebo-controlled trial. Lancet (London, England) 2004;364:685-696.

2. Kim JB, Song WH, Park JS, Youn TJ, Park YH, Kim SJ, Ahn SG, Doh JH, Cho YH and Kim JW. A randomized, open-label, parallel, multi-center Phase IV study to compare the efficacy and safety of atorvastatin 10 and 20 mg in high-risk Asian patients with hypercholesterolemia. PloS one 2021;16:e0245481.

3. Rosenson RS and Underberg JA. Systematic review: Evaluating the effect of lipid-lowering therapy on lipoprotein and lipid values. Cardiovascular drugs and therapy 2013;27:465-479.

4. Khan SU, Lone AN, Khan MS, Virani SS, Blumenthal RS, Nasir K, Miller M, Michos ED, Ballantyne CM, Boden WE and Bhatt DL. Effect of omega-3 fatty acids on cardiovascular outcomes: A systematic review and meta-analysis. EClinicalMedicine 2021;38:100997.

5. Bays HE, Ballantyne CM, Braeckman RA, Stirtan WG, Doyle RT, Jr., Philip S, Soni PN and Juliano RA. Icosapent Ethyl (Eicosapentaenoic Acid Ethyl Ester): Effects Upon High-Sensitivity C-Reactive Protein and Lipid Parameters in Patients With Metabolic Syndrome. Metabolic syndrome and related disorders 2015;13:239-247.

6. Savarese G, De Ferrari GM, Rosano GM and Perrone-Filardi P. Safety and efficacy of ezetimibe: A meta-analysis. International journal of cardiology 2015;201:247-252.

7. Ballantyne CM, Houri J, Notarbartolo A, Melani L, Lipka LJ, Suresh R, Sun S, LeBeaut AP, Sager PT and Veltri EP. Effect of ezetimibe coadministered with atorvastatin in 628 patients with primary hypercholesterolemia: a prospective, randomized, double-blind trial. Circulation 2003;107:2409-2415.

8. Sabatine MS, Giugliano RP, Keech AC, Honarpour N, Wiviott SD, Murphy SA, Kuder JF, Wang H, Liu T, Wasserman SM, Sever PS and Pedersen TR. Evolocumab and Clinical Outcomes in Patients with Cardiovascular Disease. The New England journal of medicine 2017;376:1713-1722.

9. Nicholls SJ, Puri R, Anderson T, Ballantyne CM, Cho L, Kastelein JJ, Koenig W, Somaratne R, Kassahun H, Yang J, Wasserman SM, Scott R, Ungi I, Podolec J, Ophuis AO, Cornel JH, Borgman M, Brennan DM and Nissen SE. Effect of Evolocumab on Progression of Coronary Disease in Statin-Treated Patients: The GLAGOV Randomized Clinical Trial. Jama 2016;316:2373-2384.

10. Koren MJ, Kereiakes D, Pourfarzib R, Winegar D, Banerjee P, Hamon S, Hanotin C and McKenney JM. Effect of PCSK9 Inhibition by Alirocumab on Lipoprotein Particle Concentrations Determined by Nuclear Magnetic Resonance Spectroscopy. Journal of the American Heart Association 2015;4:e002224.

11. Schwartz GG, Steg PG, Szarek M, Bhatt DL, Bittner VA, Diaz R, Edelberg JM, Goodman SG, Hanotin C, Harrington RA, Jukema JW, Lecorps G, Mahaffey KW, Moryusef A, Pordy R,

Quintero K, Roe MT, Sasiela WJ, Tamby JF, Tricoci P, White HD and Zeiher AM. Alirocumab and Cardiovascular Outcomes after Acute Coronary Syndrome. The New England journal of medicine 2018;379:2097-2107.

12. Stroes E, Guyton JR, Lepor N, Civeira F, Gaudet D, Watts GF, Baccara-Dinet MT, Lecorps G, Manvelian G and Farnier M. Efficacy and Safety of Alirocumab 150 mg Every 4 Weeks in Patients With Hypercholesterolemia Not on Statin Therapy: The ODYSSEY CHOICE II Study. Journal of the American Heart Association 2016;5:e003421.

13. Ray KK, Landmesser U, Leiter LA, Kallend D, Dufour R, Karakas M, Hall T, Troquay RP, Turner T, Visseren FL, Wijngaard P, Wright RS and Kastelein JJ. Inclisiran in Patients at High Cardiovascular Risk with Elevated LDL Cholesterol. The New England journal of medicine 2017;376:1430-1440.

14. Tsimikas S, Karwatowska-Prokopczuk E, Gouni-Berthold I, Tardif JC, Baum SJ, Steinhagen-Thiessen E, Shapiro MD, Stroes ES, Moriarty PM, Nordestgaard BG, Xia S, Guerriero J, Viney NJ, O'Dea L and Witztum JL. Lipoprotein(a) Reduction in Persons with Cardiovascular Disease. The New England journal of medicine 2020;382:244-255.

15. O'Donoghue ML, Rosenson RS, Gencer B, López JAG, Lepor NE, Baum SJ, Stout E, Gaudet D, Knusel B, Kuder JF, Ran X, Murphy SA, Wang H, Wu Y, Kassahun H and Sabatine MS. Small Interfering RNA to Reduce Lipoprotein(a) in Cardiovascular Disease. The New England journal of medicine 2022;387:1855-1864.

16. Nissen SE, Wang Q, Nicholls SJ, Navar AM, Ray KK, Schwartz GG, Szarek M, Stroes ESG, Troquay R, Dorresteijn JAN, Fok H, Rider DA, Romano S, Wolski K and Rambaran C. Zerlasiran-A Small-Interfering RNA Targeting Lipoprotein(a): A Phase 2 Randomized Clinical Trial. Jama 2024;332:1992-2002.

17. Ray KK, Bays HE, Catapano AL, Lalwani ND, Bloedon LT, Sterling LR, Robinson PL and Ballantyne CM. Safety and Efficacy of Bempedoic Acid to Reduce LDL Cholesterol. The New England journal of medicine 2019;380:1022-1032.

18. Nissen SE, Lincoff AM, Brennan D, Ray KK, Mason D, Kastelein JJP, Thompson PD, Libby P, Cho L, Plutzky J, Bays HE, Moriarty PM, Menon V, Grobbee DE, Louie MJ, Chen CF, Li N, Bloedon L, Robinson P, Horner M, Sasiela WJ, McCluskey J, Davey D, Fajardo-Campos P, Petrovic P, Fedacko J, Zmuda W, Lukyanov Y and Nicholls SJ. Bempedoic Acid and Cardiovascular Outcomes in Statin-Intolerant Patients. The New England journal of medicine 2023;388:1353-1364.

19. Gaudet D, Greber-Platzer S, Reeskamp LF, Iannuzzo G, Rosenson RS, Saheb S, Stefanutti C, Stroes E, Wiegman A, Turner T, Ali S, Banerjee P, Drewery T, McGinniss J, Waldron A,

George RT, Zhao XQ, Pordy R, Zhao J, Bruckert E and Raal FJ. Evinacumab in homozygous familial hypercholesterolaemia: long-term safety and efficacy. European heart journal 2024;45:2422-2434.

20. Bergmark BA, Marston NA, Bramson CR, Curto M, Ramos V, Jevne A, Kuder JF, Park JG, Murphy SA, Verma S, Wojakowski W, Terra SG, Sabatine MS and Wiviott SD. Effect of Vupanorsen on Non-High-Density Lipoprotein Cholesterol Levels in Statin-Treated Patients With Elevated Cholesterol: TRANSLATE-TIMI 70. Circulation 2022;145:1377-1386.

21. Rosenson RS, Gaudet D, Hegele RA, Ballantyne CM, Nicholls SJ, Lucas KJ, San Martin J, Zhou R, Muhsin M, Chang T, Hellawell J and Watts GF. Zodasiran, an RNAi Therapeutic Targeting ANGPTL3, for Mixed Hyperlipidemia. The New England journal of medicine 2024;391:913-925.

22. Ray KK, Oru E, Rosenson RS, Jones J, Ma X, Walgren J, Haupt A, Verma S, Gaudet D, Nicholls SJ and Ruotolo G. Durability and efficacy of solbinsiran, a GalNAc-conjugated siRNA targeting ANGPTL3, in adults with mixed dyslipidaemia (PROLONG-ANG3): a double-blind, randomised, placebo-controlled, phase 2 trial. Lancet (London, England) 2025;405:1594-1607.

23. Ballantyne CM, Gaudet D, Rosenson RS, Hegele RA, Zhou R, Melquist S, Hellawell J and Leeper NJ. Effect of Targeting ApoC-III With Plozasiran on Lipoprotein Particle Size and Number in Hypertriglyceridemia. Journal of the American College of Cardiology 2025;85:1839-1854.

24. Gaudet D, Pall D, Watts GF, Nicholls SJ, Rosenson RS, Modesto K, San Martin J, Hellawell J and Ballantyne CM. Plozasiran (ARO-APOC3) for Severe Hypertriglyceridemia: The SHASTA-2 Randomized Clinical Trial. JAMA cardiology 2024;9:620-630.

25. Bergmark BA, Marston NA, Prohaska TA, Alexander VJ, Zimerman A, Moura FA, Kang YM, Weinland J, Murphy SA, Goodrich EL, Zhang S, Li D, Banach M, Stroes E, Lu MT, Tsimikas S, Giugliano RP and Sabatine MS. Targeting APOC3 with Olezarsen in Moderate Hypertriglyceridemia. The New England journal of medicine 2025;393:1279-1291.

26. Karwatowska-Prokopczuk E, Tardif JC, Gaudet D, Ballantyne CM, Shapiro MD, Moriarty PM, Baum SJ, Amour ES, Alexander VJ, Xia S, Otvos JD, Witztum JL and Tsimikas S. Effect of olezarsen targeting APOC-III on lipoprotein size and particle number measured by NMR in patients with hypertriglyceridemia. Journal of clinical lipidology 2022;16:617-625.

27. Nicholls SJ, Nelson AJ, Ditmarsch M, Kastelein JJP, Ballantyne CM, Ray KK, Navar AM, Nissen SE, Harada-Shiba M, Curcio DL, Neild A, Kling D, Hsieh A, Butters J, Ference BA, Laufs U,

Banach M, Mehran R, Catapano AL, Huo Y, Szarek M, Balinskaite V and Davidson MH. Safety and Efficacy of Obicetrapib in Patients at High Cardiovascular Risk. The New England journal of medicine 2025;393:51-61.
28. Bhatt DL, Bays HE, Miller M, Cain JE, 3rd, Wasilewska K, Andrawis NS, Parli T, Feng S, Sterling L, Tseng L, Hartsfield CL, Agollah GD, Mansbach H and Kastelein JJP. The FGF21 analog pegozafermin in severe hypertriglyceridemia: a randomized phase 2 trial. Nature medicine 2023;29:1782-1792.
